# Supplementary material for: Inactivation of the ATMIN/ATM pathway protects against glioblastoma formation
Source: eLife. 2016 Mar 17;5:e08711. doi: 10.7554/eLife.08711 (PMC4811777; doi:10.7554/eLife.08711)
Supplement: Supplementary file 1. — DOI: http://dx.doi.org/10.7554/eLife.08711.036 [file elife-08711-supp1.docx]

**Supplementary File 1. Causes of death for experimental cohorts in Figures 1 and 2.**

| ID | *Atmin* | *Trp53* | *Nestin-Cre* | Age at sacrifice (days) | Brain  tumor | Type |
| --- | --- | --- | --- | --- | --- | --- |
| 212797 | *f/f* | *f/f* | positive | 431 | No | Non-tumor-related |
| 224489 | *f/f* | *f/f* | positive | 408 | No | Non-tumor-related |
| 209418 | *f/f* | *f/f* | positive | 311 | Yes | Glioblastoma |
| 209416 | *f/f* | *f/f* | positive | 290 | No | Non-tumor-related |
| 220984 | *f/f* | *f/f* | positive | 254 | No | Non-tumor-related |
| 280539 | *f/f* | *f/f* | positive | 203 | No | Pancreatic tumor |
| 227068 | *f/f* | *f/f* | positive | 290 | Yes | Glioblastoma |
| 209419 | *f/f* | *f/f* | positive | 407 | Yes | Glioblastoma |
| 131839 | +/+ | *f/f* | positive | 252 | Yes | Glioblastoma |
| 107684 | +/+ | *f/f* | positive | 252 | Yes | Anaplastic astrocytoma |
| 154755 | +/+ | *f/f* | positive | 295 | Yes | Glioblastoma |
| 128004 | +/+ | *f/f* | positive | 248 | Yes | Glioblastoma |
| 174427 | +/+ | *f/f* | positive | 329 | Yes | Glioblastoma |
| 116299 | +/+ | *f/f* | positive | 233 | Yes | Anaplastic astrocytoma |
| 148310 | +/+ | *f/f* | positive | 266 | Yes | Glioblastoma |
| 136404 | +/+ | *f/f* | positive | 291 | No | Non-tumor-related |
| 142561 | +/+ | *f/f* | positive | 209 | No | Non-tumor-related |
| 127998 | +/+ | *f/f* | positive | 289 | Yes | Low-grade fibrillary astrocytoma |
| 122126 | +/+ | *f/f* | positive | 311 | Yes | Low-grade fibrillary astrocytoma |
| 122132 | +/+ | *f/f* | positive | 231 | Yes | Anaplastic astrocytoma |
| 122137 | +/+ | *f/f* | positive | 285 | Yes | Glioblastoma |
| 261625 | +/+ | *f/f* | positive | 149 | Yes | Glioblastoma |
| 243131 | +/+ | *f/f* | positive | 271 | Yes | Anaplastic astrocytoma |
| 273525 | +/+ | *f/f* | positive | 271 | Yes | Glioblastoma |
| 267266 | +/+ | *f/f* | positive | 261 | No | Osteosarcoma |

N.B. Excludes animals alive at endpoint (6 from *Atmin^ΔN^; p53^ΔN^*, 0 from *p53^ΔN^*, 9 from *Atmin^ΔN^* cohorts)
