# Supplementary material for: Inactivation of the ATMIN/ATM pathway protects against glioblastoma formation
Source: eLife. 2016 Mar 17;5:e08711. doi: 10.7554/eLife.08711 (PMC4811777; doi:10.7554/eLife.08711)
Supplement: Supplementary file 2. — DOI: http://dx.doi.org/10.7554/eLife.08711.037 [file elife-08711-supp2.docx]

**Blake et al., Supplementary file 1**

Primer sequences

Mm Atmin fwd 5’- ccgagtgggacctgaaaag

Mm Atmin rev 5’- cgcacgtacactggaaggt

Mm Pdgfra fwd 5’- gcgagtttaatgtttatgccttg

Mm Pdgfra rev 5’- ggcacaggtcaccacgat

Mm Pdgfrb fwd 5’- tcaagctgcaggtcaatgtc

Mm Pdgfrb rev 5’- ccattggcagggtgactc

Mm Pdgfa fwd 5’- gatgaggacctgggcttg

Mm Pdgfa rev 5’- gatcaactcccggggtatct

Mm Pdgfb fwd 5’- cggcctgtgactagaagtcc

Mm Pdgfb rev 5’- gagcttgaggcgtcttgg

Mm Mekk1 fwd 5’- gcagttttaaccttactcattttgg

Mm Mekk1 rev 5’- agttccattccaaacacctga

Mm Igf2bp3 fwd 5’- aaacagctttctcgctttgc

Mm Igf2bp3 rev 5’- tccgcactttagcatctggt

Mm Cpne8 fwd 5’- tgtccttcttggtggaaaca

Mm Cpne8 rev 5’- gtgaagttgatctgcgttcct

Mm Actin fwd 5’- tctttgcagctccttcgttg

Mm Actin rev 5’- acgatggaggggaatacagc

Hs Pdgfra fwd 5’- ccacctgagtgagattgtgg

Hs Pdgfra rev 5’- tcttcaggaagtccaggtgaa

Hs Actin fwd 5’- gctacgagctgcctgacg

Hs Actin rev 5’- ggctggaagagtgcctca
